# Relationship between self-efficacy and patient knowledge on adherence to oral contraceptives using the Morisky Medication Adherence Scale (MMAS-8)

**DOI:** 10.1186/s12978-017-0374-6

**Published:** 2017-09-06

**Authors:** Daniel Tomaszewski, Benjamin D. Aronson, Margarette Kading, Donald Morisky

**Affiliations:** 1 Chapman University School of Pharmacy, 9401 Jeronimo Rd, Irvine, California, 92618 USA; 2 Ohio Northern University Raabe College of Pharmacy, 525 S Main St, Ada, OH 45810 USA; 3 College of Pharmacy, University of Minnesota, 232 Life Sciences, 1110 Kirby Dr, Duluth, MN 55812 USA; 4 UCLA Fielding School of Public Health, 650 Charles E. Young Dr. South, 16-035 Center for Health Sciences, Los Angeles, CA 90095-1772 USA

**Keywords:** Oral contraceptive adherence, Self-efficacy, Knowledge, Oral contraceptive pill, College students

## Abstract

**Background:**

Preconception care, including family planning, is a vital component of healthcare for women of reproductive age. An average female spends the majority of her reproductive life trying to prevent a pregnancy. In order to prevent unintended pregnancy, women often rely on the use of hormonal contraceptives. In the United States, the majority of hormonal contraceptive users are prescribed oral contraceptive pills (OCPs). Reduced adherence to OCPs decreases their ability to prevent pregnancy. The study aimed to measure OCP adherence among female college students, and explore the relationship between OCP adherence, knowledge, and self-efficacy.

**Methods:**

This cross-sectional study recruited a random sample of female college students to participate in an online survey. OCP adherence was based on the 8-item Morisky Medication Adherence Scale (MMAS-8). Secondary reporting of medication adherence included participant reports of the number of missed OCP doses in the previous month and typical month of use.

**Results:**

Of the 5000 invited, 1559 (31.3%) completed the survey. Of those responding, 670 (41.3%) reported use of OCPs. A total of 293 (44.3%) OCP users met criteria for low adherence, 241 (36.4%) met criteria for medium adherence, and 128 (19.3%) met criteria for high adherence. Those with high adherence had higher self-efficacy (*P* < 0.001) and perceived knowledge (*p* < 0.001). After controlling for other factors, self-efficacy (b = .37) and perceived knowledge (b = .09) remained associated with OCP adherence.

**Conclusion:**

Less than 20% of respondents met the criteria for high adherence to OCPs. Self-efficacy and knowledge were associated with higher OCP adherence. Targeted interventions from healthcare providers, health educators, and other adherence related media to increase the knowledge and self-efficacy of patients using OCPs may improve adherence rates. Additional research is needed to evaluate the impact of innovative interventions focused on social and behavioral patient factors, like knowledge and self-efficacy, on adherence to OCPs.

**Plain English summary**

A large portion of women rely upon oral contraceptive pills (OCPs) as a part of their family planning strategy. For OCPs to work effectively at preventing pregnancies, women must consistently take them. In other words, OCPs require good adherence. In this study women attending college at one university were recruited to answer questions in an online survey to detect any problems with OCP adherence, and determine if self-efficacy or knowledge influence OCP adherence.

The women that participated in the online survey were asked if they used OCPs. Those who used OCPs were asked about their self-efficacy, or feeling that they could confidently take their birth control correctly, knowledge of their OCP, and adherence in taking their OCP. In addition, the women in this study provided their age, relationship status, reason for OCP use, and past use of emergency contraception.

A total of 670 women, or just over 40% of those in this study, used OCPs. Over 50% of these women missed at least one dose of their OCP in the month prior to the survey. Adherence to OCPs was related to both self-efficacy and knowledge.

In sum, there is room to improve OCP adherence in women attending college. Increasing knowledge about OCPs and building self-efficacy may be effective strategies to improve OCP adherence.

## Background

Preconception care, including family planning, is a vital component of healthcare for women of reproductive age. Unsuccessful pregnancy prevention efforts have the potential to lead to unintended pregnancies. The consequences of an unintended pregnancy, which include those that were mistimed and unwanted, include impacts to the fetus, the mother, and society. Fetal impacts include a delay in receiving necessary prenatal supplements, potential for exposure to environmental risks (including alcohol, nicotine, or other drugs), and increased potential for birth defects and low birth weight [1]. Additionally, children resulting from unintended pregnancies have been shown to experience poor mental and physical health and have lower educational attainment and more behavioral health issues in their teens [2]. Mothers of unintended pregnancies have been shown to be at increased risks of adverse effects from delays in prenatal care, maternal depression, and reduction in income potential [3]. Lastly, a significant burden has been placed on society by unintended pregnancies. Estimates suggest that unintended pregnancies cost the United States $21 billion in 2010, which includes the cost of births, abortions, and miscarriages from unintended pregnancies [4].

Although only 8% of U.S. women report unmet family planning needs and 99.1% of sexually active women report having used some form of contraceptive, there remains significant disparities in pregnancy prevention [5, 6]. Prior to 2011 there had been nearly no change over the last 30 years in pregnancies that were reported as unintended when conceived (49%) or births that were reported to be unintended (37%) [7–9]. This is particularly troubling, as the availability of contraceptives has improved over this same timeframe [9]. Although these rates dropped in 2011 to 45% and 36%, respectively, there remains a considerable concern over the high unintended rate and the prevention of unintended pregnancy continues to be a key focus of the public health system in the United States [1].

Consistent and correct use of modern contraception has a dramatic impact on unintended pregnancies. Only 68% of women that are at risk for an unintended pregnancy report correct and consistent use of contraception, and these women account for only 5% of unintended pregnancies [10]. Estimates suggest that non-adherence may be directly the cause of approximately 20% of unintended pregnancies each year [11]. For oral hormonal contraceptives (also known as “the pill” or oral contraceptive pill, OCP), perfect use requires users to take every dose within the same 4-h window each day. Those who are not able to maintain perfect use, or are non-adherent to their contraception method of choice, reduce the effectiveness rates considerably [11]. Perfect adherence has been shown to prevent nearly all unintended pregnancies (<1% failure rate), compared to a nearly 9% failure rate with typical use (i.e., inconsistent or incorrect use) [12, 13].

In this study, less than perfect adherence is referred to as poor adherence, or nonadherence. Past estimates of OCP nonadherence range from 25% not taken within the “window of hormonal safety” [14], to approximately 33–52% of women missing 1 or more pill per cycle [12, 15–17], with over 20% missing 2 or more pills per cycle [16]. Even in an oral contraceptive clinical trial, the rate of nonadherence (labelled noncompliance in the study) was 17% [18]. It is important to consider potential causes of non-adherence and interventions that may appropriately target these causes.

Previous research has suggested a number of factors associated with poor OCP adherence, but the research is limited overall [19–21]. Previously identified correlates of OCP adherence include the establishment of a pill taking routine, information seeking habits, and race and ethnicity [19, 20]. In addition, one study showed higher levels of patient reported perceived behavioral control and the presence of coping strategies for non-adherence were positively associated with adherence to OCPs [21].

Given the limited data regarding influential factors of OCP adherence, this study aims to investigate several possible determinants. The primary objectives of this study are to: 1) determine self-reported adherence to OCPs based upon a validated measure, 2) explore demographic differences in adherence rates, 3) examine the relationship between self-efficacy and perceived knowledge on adherence.

## Methods

This cross-sectional research was conducted using an online survey platform, Qualtrics®. The survey was electronically distributed to participants using their preferred email. The online survey platform allowed complex skip patterns and flow logic to be employed, whereby a respondent’s earlier choice in the survey determined the question displayed, and also the specific OCP product referenced in the question. Participants not currently using any form of hormonal contraceptive were directed to answer a separate set of questions designed to gather basic demographics, involvement in risky behaviors, and method of pregnancy prevention used. The University of Minnesota and Chapman University Institutional Review Boards approved this study.

### Participants

This study was conducted at the University of Minnesota, a public research university in the Midwestern United States with approximately 50,000 thousand enrolled students, of which over 25,000 are female. A simple random sample of 5000 female students was obtained from the University of Minnesota’s Office of Institutional Research. This sample was limited to the list of degree-seeking females with full-time enrollment status aged 18 and older. Students who had suppressed their contact information were excluded from the panel. A total of 5000 email invitations were sent to potential participants, 14 of which were returned as undeliverable. One reminder e-mail was sent 7 days after the initial e-mail to selected individuals that had not yet responded. In sum, 1750 individuals opened the survey, 1623 consented to participate, and 1559 completed the survey. The survey completion rate of invited individuals who received the invitation email was 31.3% (1559/4986). Only those taking OCPs were included in the analyses below (*n* = 670).

### Measures

#### OCP Adherence

Adherence to OCPs was measured using the 8-item Morisky Medication Adherence Scale (MMAS-8) [22]. This scale is extensively used in survey research as a proxy for medication adherence, and has shown concurrent validity with non-humanistic markers of adherence [22–25]. The items in the scale ask participants questions about their medication-related behaviors and causal indicators, for example, “Do you sometimes forget to take your oral contraceptive?” The first seven items have a yes/no response format. The final item, “How often do you have difficulty remembering to take your oral contraceptive?” A five-item Likert scale was used to measure item 8.. Items were summed to provide a continuous measure of self-reported adherence ranging from 0 to 8, with higher scores indicating higher adherence. No participants had missing data on more than one MMAS-8 item, thus all met the 75% eligibility criterion for the scale. Per standard scoring procedures, missing data on MMAS-8 items were imputed by using the median value from those participants who met the eligibility criteria. Cut points were used to categorize high (sum score = 8), medium (sum score 6 to <8), and low (sum score < 6) adherence based on previously established categories [22]. In the original study of patients with high blood pressure, high adherence was associated with blood pressure control, which further demonstrated the criterion-related validity of the MMAS-8 in measuring adherence [22]. In order to compare the present findings to prior studies, we also asked how many days participants forgot to take their OCP in a typical month, and then more specifically last month. Response options were Never, 1 time per month, 2–3 times per month, 4–5 times per month, and 6 or more times per month. Self-report of missed doses was used to describe the sample, and compared by adherence category.

#### Correlates of OCP Adherence

Perceived knowledge of OCP being used was measured using one item. Perceived knowledge is defined here as a self-assessment of knowledge, or in other words, what each respondent thinks they know about their OCP. Participants were asked: How would you rate your knowledge of your hormonal contraceptive, on a scale from 1 to 10, where 1 means “I know nothing” and 10 means “I know everything.” Responses were recoded to be grounded at zero (i.e., ranging from 0 to 9), with a higher score equating to higher perceived knowledge. Self-efficacy of using OCP correctly was measured with one item. Here, self-efficacy is defined as the belief that one can successfully perform a behavior [26], namely in this study, correctly use her OCP. We measured self-efficacy by asking individuals to indicate their level of agreement on a 5-point Likert-type scale with the statement “I feel confident that I use my contraceptive correctly.” The responses were coded from 0 (‘Strongly Disagree’) to 4 (‘Strongly Agree’).

#### Covariates

Participants reported their age, relationship status, reason for OCP use, and past emergency contraceptive use. Age was collected as a continuous variable. Participants indicated their current relationship status, and we dichotomized responses as in a committed relationship (married, committed dating relationship, engaged) or not in a committed relationship (single, separated, widowed, divorced). Participants were asked to report the reasons why they were using OCP (i.e., pregnancy prevention, help regulate menses, reduce symptoms associated with menses, improving acne, treatment of premenstrual dysphoric disorder, or other), which was used to create a new variable dichotomized by those indicating that pregnancy prevention was one of the reasons they were using OCP (1), and those that did not (0).

#### Statistical analysis

We first examined the descriptive statistics for all study variables. To examine potential differences in participant characteristics and bivariate relationships between study variables, we used ANOVA for continuous variables and chi square test for categorical variables. The alpha value for statistical significance was set at 0.05, with a Bonferroni correction applied for comparisons of continuous variables between adherence categories. We used ordinary least squares regression to determine the relationship between perceived knowledge, self-efficacy, and medication adherence after controlling for age, relationship status, and use of OCP for pregnancy prevention. List-wise deletion of cases with missing data resulted in 650 total observations for regression analysis.

## Results

The overall sample contained 670 OCP users (41.9%), 228 other contraceptive users (14.3%), and 700 non-users (43.8%). Of the OCP users, 293 (44.3%) met criteria for low adherence, 241 (36.4%) met criteria for medium adherence, and 128 (19.3%) met criteria for high adherence (Table 1). The mean score for OCP users’ reported knowledge of their OCP was 6.03 (scored from 0 to 9), signifying a potential knowledge gap. Conversely, a majority of participants reported higher rates of self-efficacy, with a mean score of 3.43 (scored 0 to 4). Additional participant data and demographic characteristics are shown in Table 1.

**Table 1:** Demographic characteristics of participants using OCPs

	Mean/% (S.D.)
Age	21.95 (4.1)
Years in college	3.42 (2.1)
Relationship status	
In a relationship	61.2%
Not in a relationship	38.8%
Primary reason(s) for using hormonal contraception^a^	
Pregnancy prevention	84.6%
Regulation of menses	71%
Reduce symptoms associated with menses	52.7%
Improving acne	35.4%
Treatment of Premenstrual Dysphoric Disorder	2.5%
Other	3.7%
Sexual orientation	
Heterosexual	92.5%
Gay/Lesbian	0.8%
Bisexual	4.7%
Unsure	2%
Typical month missed doses	
Never	37.8%
1 time per month	39.6%
2–3 times per month	18.6%
4–5 times per month	2.7%
6 or more times per month	1.2%
Last month missed doses	
Never	46.9%
1 time per month	31.2%
2–3 times per month	17.9%
4–5 times per month	3.3%
6 or more times per month	0.8%
Mean perceived knowledge (range 0 to 9)	6.03 (1.5)
Mean perceived self-efficacy (range 0 to 4)	3.43 (0.6)

^a^Participants could select multiple responses; OCPs oral contraceptive pills; S.D. standard deviation

Table 2 provides a comparison of demographics and correlates of adherence by OCP adherence category. Significant differences were found between groups for relationship status (*P* = 0.016), with the proportion in a relationship highest among the high adherence group. There were significant differences in perceived knowledge (*P* < 0.001) and perceived self-efficacy (*P* < 0.001). The low adherence group had significantly lower perceived knowledge compared to the medium (*P* = 0.002) and high (*P* < 0.001) adherence groups. Additionally, the low adherence group had significantly lower perceived self-efficacy compared to the medium and high groups (*P* < 0.001 for both), and the medium group had significantly lower self-efficacy than the high group (*P* = 0.028).

**Table 2:** Demographic characteristics and correlates of adherence by OCP adherence category

	Low adherence	Medium adherence	High adherence	*P*
Overall (%)	44.3%	36.4%	19.3%	n/a
Age (mean)^a^	21.8	21.9	22.4	.346
Years in college (mean)^a^	3.4	3.3	3.6	.559
In a relationship (%)^B^	55.9%	61.7%	71.9%	.009
OCP for pregnancy prevention (%)^b^	84.2%	84.2%	87.5%	.652
Perceived knowledge (mean)^a^	5.74	6.18	6.40	<.001
Perceived self-efficacy (mean)^a^	3.21	3.54	3.71	<.001
2 or more missed pills typical month^b^	45.9%	5.8%	0.8%	<.001
2 or more missed pills last month^b^	42.7%	8.3%	0%	<.001

^a^ANOVA

^b^chi-square test

Use of the ©MMAS is protected by US copyright laws. Permission for use is required. A Licensure agreement is available from: Donald E. Morisky, ScD, ScM, MSPH, Professor, Department of Community Health Sciences, UCLA School of Public Health, 650 Charles E. Young Drive South, Los Angeles, CA 90095–1772, dmorisky@ucla.edu

Results of ordinary least squares regression are shown in Table 3. After controlling for age, relationship status, and use of OCP for pregnancy prevention, perceived knowledge (beta = .08; *P* = .032) and self-efficacy (beta = .37; *P* < .001) were both positively related to OCP adherence scores using the MMAS-8. The R^2^ of the regression model was 0.16.

**Table 3:** Results of ordinary least squares regression of OCP adherence score

	Unstandardized coefficient (B)	Standard error	Standardized coefficient (beta)	*P*
Constant	2.45	.46		.000
Age	.00	.01	−.02	.641
In a relationship	.19	.12	.06	.116
OCP for pregnancy prevention	−.06	.16	−.01	.725
Perceived knowledge	.09	.04	.08	.032
Perceived self-efficacy	.93	.10	.37	.000
Model adjusted R^2^	.16			

## Discussion

This study determined the rate of OCP adherence, explored demographic differences in adherence rates, and examined the relationship between self-efficacy and perceived knowledge on adherence for a sample of women enrolled at a large Midwest university. According to the MMAS-8, 80.7% of OCP users exhibited some degree of non-adherence, or in other words less than perfect adherence, higher than past estimates of non-adherence to OCPs [12–18]. The report of missed doses in an average/typical month in this study was also higher than previously reported estimates (62% reported missing at least 1 dose per month, compared to 33–52% reported in previous research [12, 15–17]).

Several differences were noted between adherence groups when categorized by the MMAS-8. While there was no difference in age, years in college, or use of OCP for pregnancy prevention, a higher proportion of those in the high adherence group reported being in a relationship. This latter finding is consistent with other research demonstrating that women in consistent relationships are more likely to use effective contraception when compared to women in casual relationships [27]. As expected, proportionally fewer individuals in the high adherence group reported missing 2 or more doses in both a typical month and last month, and the high adherence group exhibited a higher mean perceived knowledge and higher mean perceived self-efficacy when compared to the low adherence group.

After controlling for demographic factors, perceived knowledge and self-efficacy remained positively related to OCP adherence. These findings, while cross-sectional, suggest that knowledge and self-efficacy could be useful targets for future interventional research focused on improving OCP adherence. This is supported by prior work that has shown the amount of accompanying educational material read and understood was related to adherence [19]. Additionally, a study by Molloy and colleagues established the relationship between perceived behavioral control, a concept similar to self-efficacy, and medication adherence among OCP users [21]. These findings in OCP adherence are consistent with a cadre of prior literature that established the relationship between self-efficacy on medication adherence for other disease states [28, 29].

Current recommendations for counseling adolescents about contraception include assessment of self-efficacy toward a contraceptive method [30]. This recommendation is also shared within the CDC’s and U.S. Office of Population Affairs’ family planning recommendations, which recommends that the provider evaluates potential barriers to continued appropriate use of the contraception being considered [31]. These recommendations encourage the provision of counseling services to improve patient knowledge, but also highlight the importance of evaluating social-behavioral factors, including patient confidence in ability to use consistently and correctly [31]. Our findings support these recommendations.

### Limitations

Although this study provides insight on factors influencing OCP adherence, the findings must be placed in context of the limitations. First, the use of a cross-sectional method to collect data does not allow causality to be inferred from the results. In addition, the methodology employed in this study could lead to volunteer bias, and potential recall bias. Use of the MMAS-8 has historically focused on measuring adherence to medications related to chronic or long-term infectious conditions, and though OCP use is similar to other chronic medications (i.e. medication must be taken daily), it is unclear if the MMAS is the most appropriate medication adherence scale to use. Previous research has shown the ability to tailor the MMAS-8 to unique disease states and dosage forms, such as, asthma inhalants, injectable insulins for diabetes, and oral osteoporosis medications dosed less frequently than daily [32]. In this study, self-report of missed doses was related to the MMAS-8 adherence categories, providing evidence for its use in OCP research. Further research and modification to the original scale may be warranted to develop an optimal scale. This study’s cohort had a higher education level compared to the general population in the U.S., limiting the generalizability of these findings to the overall U.S. population. It is concerning that in this highly educated sample such high levels of missed OCP doses were found. Lastly, the measures of perceived knowledge and self-efficacy used in this study were single-item indicators. While these indicators could be helpful clinical tools in terms of ease of administration, answers do not specifically indicate what knowledge is lacking or what elements of self-efficacy are low. This limits the ability to more specifically examine possible reasons for OCP nonadherence, and design interventions to remedy these gaps. However, low perceived knowledge or self-efficacy reported by a patient could prompt a clinician to delve into tailored counseling to ameliorate knowledge gaps and improve self-efficacy.

## Conclusion

Based upon the adherence measure used, only 20% of the women in this study met criteria for a high level of OCP adherence. The findings from multivariable analysis affirms the relationship between self-efficacy and perceived knowledge on OCP adherence. We are not aware of any other study that has linked perceived knowledge of OCPs to OCP adherence. These findings highlight the importance of patient knowledge and self-efficacy and the need to target these factors to improve adherence. Healthcare practitioners, health educators, and other adherence related media may need to consider focusing their attention to efforts aimed at ensuring proper patient knowledge and a better sense of self-efficacy in managing OCP use. More research is necessary to determine the best measure of OCP adherence and effective approaches to improve self-efficacy and knowledge of patients to their OCP. Additionally, future research needs to measure the impact of these approaches on adherence to OCPs and reduction in unintended pregnancies.

## Abbreviations

ANOVA: Analysis of Variance
MMAS-8: Morisky Medication Adherence Scale (8-item)
OCP: Oral Contraceptive Pill
